# Systemic Perturbation of the ERK Signaling Pathway by the Proteasome Inhibitor, MG132

**DOI:** 10.1371/journal.pone.0050975

**Published:** 2012-11-30

**Authors:** Murat Cirit, Kyle G. Grant, Jason M. Haugh

**Affiliations:** Department of Chemical and Biomolecular Engineering, North Carolina State University, Raleigh, North Carolina, United States of America; Hungarian Academy of Sciences, Hungary

## Abstract

Inhibition of the ubiquitin-proteasome protein degradation pathway has been identified as a viable strategy for anti-tumor therapy based on its broad effects on cell proliferation. By the same token, the variety of elicited effects confounds the interpretation of cell-based experiments using proteasome inhibitors such as MG132. It has been proposed that MG132 treatment reduces growth factor-stimulated phosphorylation of extracellular signal-regulated kinases (ERKs), at least in part through upregulation of dual specificity phosphatases (DUSPs). Here, we show that the effects of MG132 treatment on ERK signaling are more widespread, leading to a reduction in activation of the upstream kinase MEK. This suggests that MG132 systemically perturbs the intracellular phosphoproteome, impacting ERK signaling by reducing phosphorylation status at multiple levels of the kinase cascade.

## Introduction

Signal transduction pathways and networks direct cell responses largely through post-translational modifications, e.g., phosphorylation/dephosphorylation of their protein components. But the rates of these modifications depend in turn on the intracellular concentrations of enzymes and other regulatory proteins; thus, mechanisms governing protein synthesis and degradation are equally central to the regulation of cell signaling.

The ubiquitin-proteasome pathway is an essential quality control mechanism directing degradation of mislocated, misfolded, and damaged proteins, and, by tempering the expression levels of specific signaling proteins, it also exerts a level of control over cell physiology [1]. Poly-ubiquitinated proteins, targeted by E3 ubiquitin ligases, can be recognized and degraded by the 26S proteasome, a multi-subunit, multi-catalytic protease machine [2]. Proteasome inhibitors have shown great promise as cancer therapeutics because they impact a variety of mechanisms affecting tumor cell proliferation and survival; proteasome inhibition interferes with cell cycle progression, upregulates tumor suppressors such as p53, and diminishes activation of pro-proliferation pathways such as those controlled by NFκB and extracellular signal-regulated kinases (ERKs) [3], [4].

The mitogen-activated protein kinases (MAPKs) ERK2/MAPK1 and ERK1/MAPK3 (hereafter referred to collectively as ERK1/2) are activated by phosphorylation in a canonical Raf → MEK → ERK kinase cascade in response to most growth factors and cytokines, and ERK1/2 phosphorylate more than 150 cytosolic and nuclear substrates [5], [6]. Thus, they are master controllers of cell proliferation, differentiation, and migration. ERK signaling is inappropriately activated in a wide array of human cancers, which can be caused by an activating mutation in one of the upstream signaling proteins or through overexpression of growth factors or growth factor receptors [7], [8]. The dual specificity phosphatases (DUSPs) have been linked to dephosphorylation of ERK1/2 and other MAPKs [9], and in many contexts, DUSP expression levels are known to be regulated through the ubiquitin-proteasome degradation pathway [10]–[15]. Accordingly, cells treated with MG132 or other proteasome inhibitors exhibit higher expression of MKP3/DUSP6, an ERK1/2-specific DUSP, accompanied by lower levels of ERK phosphorylation stimulated by growth factors [12]–[14].

Considering that a host of intracellular proteins are affected by proteasome inhibition, coupled with evidence that knockdown of MKP3 expression enhances growth factor-stimulated ERK phosphorylation in some contexts [13] but not in others [16], led us to question whether or not the diminution of ERK signaling in MG132-treated cells could be attributed solely to upregulation of MKP3 and other DUSPs. In this short paper, we confirm that MG132 treatment reduces phosphorylation of ERK in fibroblasts stimulated with platelet-derived growth factor (PDGF) or basic fibroblast growth factor (FGF) and show that this is caused by two parallel effects. For a given level of MEK activation, ERK phosphorylation is reduced, consistent with the proposed upregulation of ERK phosphatase activity, but maximal MEK activation is also diminished.

**Figure 1:**
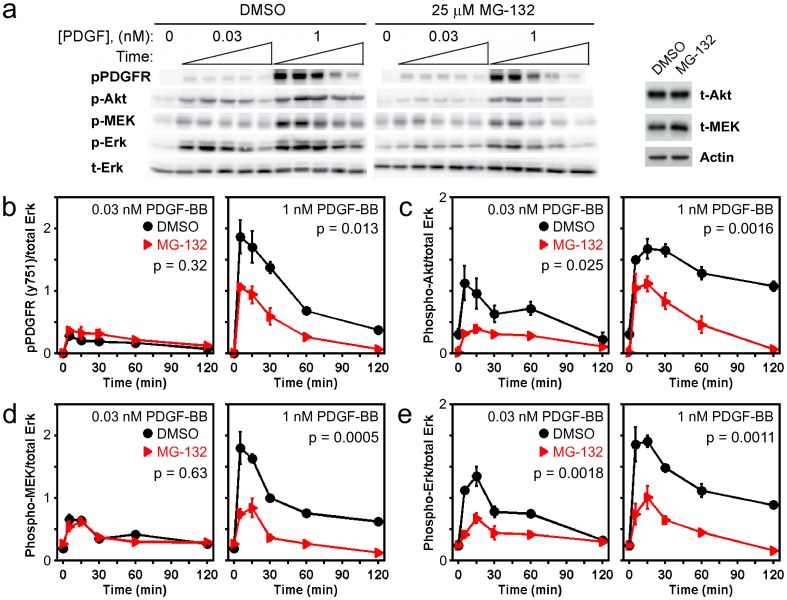
MG132 treatment depresses PDGF-stimulated phosphorylation of ERK and also reduces the activation of upstream signaling components. *a*) Immunoblot results, representative of 3 independent experiments, showing the phosphorylation kinetics of PDGF β-receptor Tyr^751^ (pPDGFR), Akt1/2/3 Ser^473^ (pAkt), MEK1/2 Ser^217^/Ser^221^ (pMEK), and ERK1/2 Thr^202^/Tyr^204^ (pERK) in cells pretreated with either DMSO or 25 µM MG132 for 6 h and then stimulated with the indicated concentration of PDGF-BB. Stimulation times are 5, 15, 30, 60, and 120 minutes. Total ERK1/2 (tERK) serves as a loading control. For each antigen, the DMSO and MG132 bands are cropped from the same gel. At right it is shown that total Akt (tAkt) protein expression is not affected by MG132 treatment, whereas total MEK1/2 (tMEK) is only modestly increased in MG132-treated cells, relative to β-actin loading control. *b-e*) Quantification of the phosphorylation kinetics represented in *a*. Each readout is normalized by total ERK and expressed as mean ± s.e.m. (*n* = 3): *b*, pPDGFR; *c*, pAkt; *d*, pMEK; *e*, pERK. The indicated *p* value for each time course is from two-way ANOVA analysis comparing MG132-treated and control measurements.

## Materials and Methods

### Reagents

Human recombinant PDGF-BB and murine recombinant FGF-2 were purchased from Peprotech (Rocky Hill, NJ). Antibodies against total ERK1/2, MEK1/2, Akt1/2/3 and MKP3 and phospho-specific antibodies against PDGF β-receptor pTyr^751^, Akt pSer^473^, ERK pThr^202^/pTyr^204^, and MEK pSer^217^/pSer^221^ were from Cell Signaling Technology (Beverly, MA). Antibodies against MKP1 were from Santa Cruz Biotechnology (Santa Cruz, CA). MG132 was purchased from Calbiochem (San Diego, CA) and aliquoted in DMSO; cells were incubated with the drug at a final concentration of 25 µM, with an equivalent concentration of DMSO (0.2% v/v) serving as a vehicle control. All tissue culture reagents were from Invitrogen (Carlsbad, CA). Unless otherwise noted, all other reagents were from Sigma-Aldrich (St. Louis, MO).

**Figure 2:**
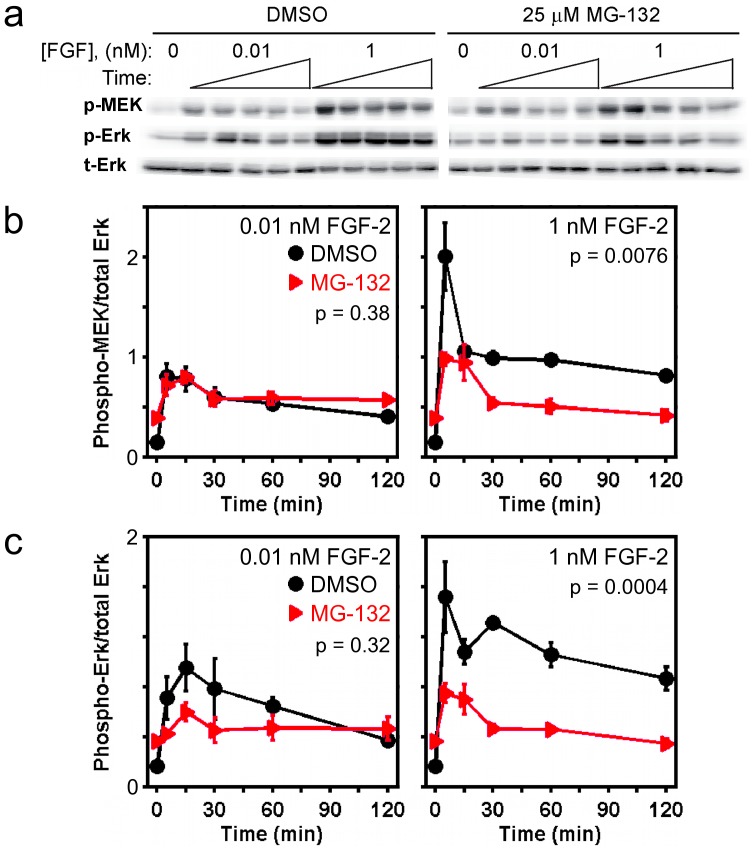
MG132 treatment reduces FGF-stimulated phosphorylation of MEK and ERK. *a*) Immunoblot results, representative of 3 independent experiments, showing the kinetics of pMEK and pERK in cells pretreated with either DMSO or 25 µM MG132 for 6 h and then stimulated with the indicated concentration of FGF-2. Stimulation times are 5, 15, 30, 60, and 120 minutes. Total ERK1/2 (tERK) serves as a loading control. For each antigen, the DMSO and MG132 bands are cropped from the same gel. *b&c*) Quantification of the MEK (*b*) and ERK (*c*) phosphorylation kinetics represented in *a*. Each readout is normalized by total ERK and expressed as mean ± s.e.m. (*n* = 3). The indicated *p* value for each time course is from two-way ANOVA analysis comparing MG132-treated and control measurements.

### Cell Culture and Immunoblotting

NIH 3T3 mouse fibroblast and HT-1080 human fibrosarcoma cell lines were acquired from American Type Culture Collection (Manassas, VA). Mouse embryonic fibroblasts, derived from pregnant CD-1 mice (Charles River Laboratories, Wilmington, MA), were isolated according to standard protocol [17] and kindly provided by the laboratory of Balaji Rao (North Carolina State University, Raleigh, NC). All cells were cultured at 37°C, 5% CO_2_ in Dulbecco’s Modified Eagle Medium supplemented with 10% fetal bovine serum, 2 mM L-glutamine, and the antibiotics penicillin and streptomycin. Cells were serum-starved for 3 hours, followed by pretreatment with MG132 or DMSO vehicle control for the time indicated. The cells were then stimulated with either PDGF-BB or FGF-2 as indicated, in the continued presence of MG132 or DMSO. Quantitative immunoblotting from detergent prepared lysates was performed using enhanced chemiluminescence, and densitometry data were normalized as described in detail previously [16], [18]. Statistical analysis of each time course was performed by two-way analysis of variance (ANOVA); in each case the null hypothesis is that MG132 treatment has no effect relative to the DMSO control.

**Figure 3:**
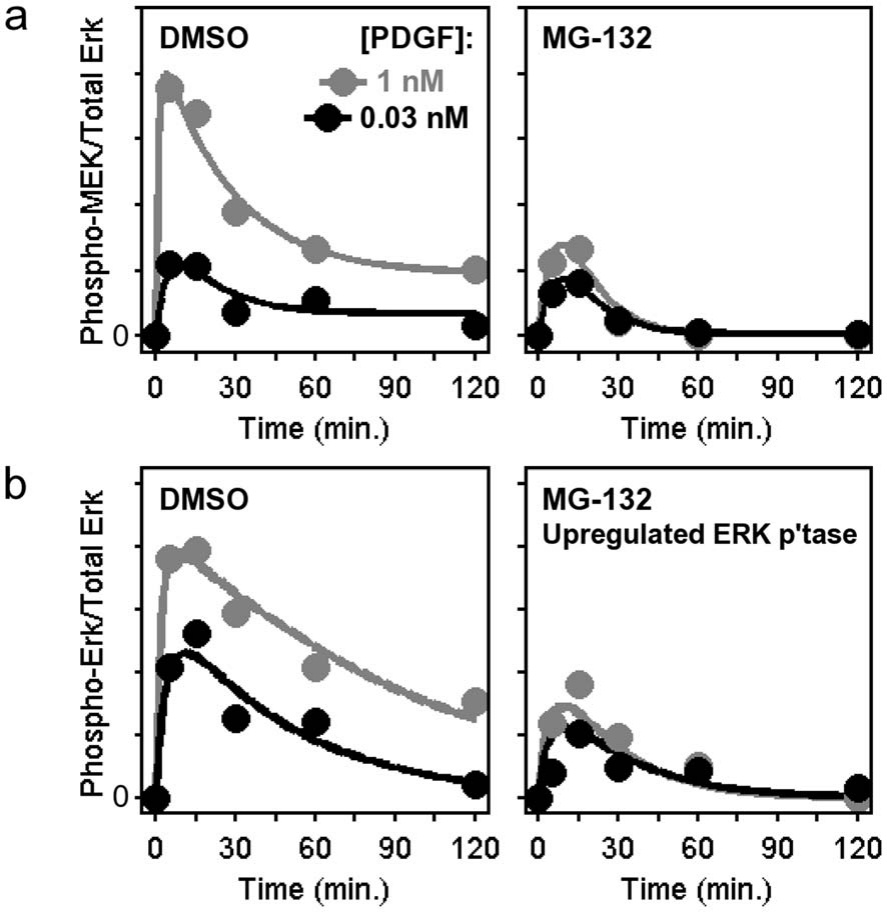
Estimate of ERK phosphatase upregulation in MG132-treated cells by computational modeling. *a*) Each time course of MEK phosphorylation in PDGF-stimulated cells from Fig. 1d was fit to a phenomenological function of time. Black, 0.03 nM PDGF; gray, 1 nM PDGF. *b*) Using the phosphorylated MEK kinetics as an input, ERK phosphorylation kinetics were globally fit to a modified Michaelis-Menten model, allowing that ERK phosphatase activity is upregulated in MG132-treated cells. The fold-upregulation is applied to both PDGF concentrations: black, 0.03 nM PDGF; gray, 1 nM PDGF.

### Kinetic Model and Computational Analysis

A semi-mechanistic model of ERK phosphorylation was developed to estimate the fold-upregulation of ERK phosphatase activity in MG132-treated cells, using the time course of MEK phosphorylation as an input. Given that MEK phosphorylation is also perturbed by MG132 treatment, our strategy was to independently fit each time course of MEK phosphorylation to a phenomenological function; then, assuming those phosphorylated MEK kinetics, ERK phosphorylation kinetics were globally fit to a modified Michaelis-Menten model (Text S1).

**Figure 4:**
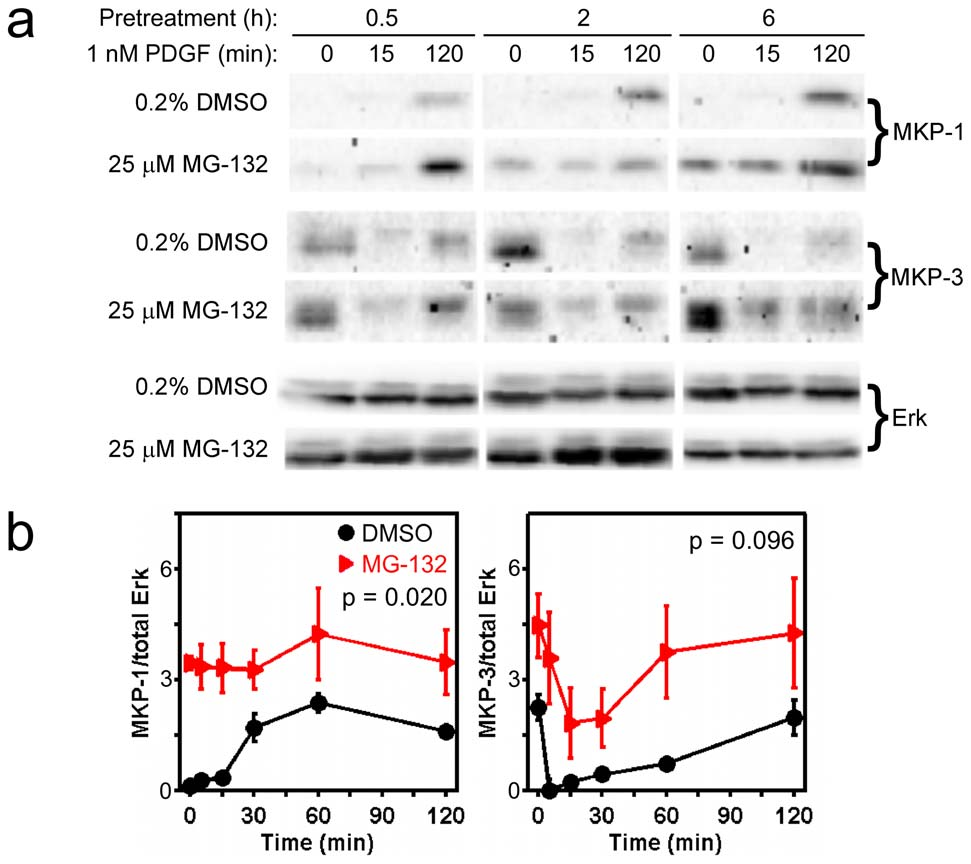
Upregulation of MKP1 and MKP3 protein levels in cells treated with MG132. *a*) Immunoblot results indicating the effect of MG132 pretreatment time on MKP1 and MKP3 upregulation in unstimulated or PDGF-stimulated NIH 3T3 cells. Total ERK1/2 levels are not significantly perturbed by MG132 treatment. The 18 bands for each antigen are cropped from the same gel and rearranged. *b*) Quantification of relative MKP1 and MKP3 expression levels as a function of PDGF (1 nM) stimulation time, comparing cells pretreated for 6 h with 25 µM MG132 or DMSO vehicle only. Each readout is normalized by total ERK and expressed as mean ± s.e.m. (*n* = 3, independent experiments). The indicated *p* value for each time course is from two-way ANOVA analysis comparing MG132-treated and control measurements.

All calculations were performed using MATLAB (MathWorks, Natick, MA). The parameter estimation approach used is as described in detail previously [16], [19]. Briefly, it uses a Markov chain Monte Carlo/simulated annealing-based algorithm to generate a large (*n* = 10^4^) ensemble of “good” parameter sets rather than one “best” fit. After compiling the ensemble, the model output is recalculated for each parameter set, and at each time point, an ensemble mean and standard deviation are calculated.

**Figure 5:**
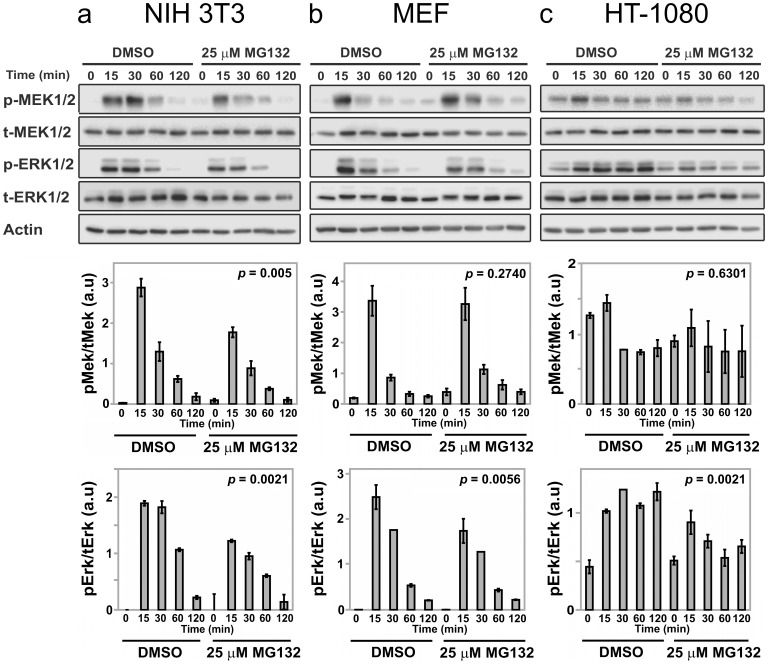
Sensitivities of PDGF-stimulated MEK and ERK phosphorylation to MG132 treatment in different mesenchymal cell backgrounds. Immunoblot results, each representative of 3 independent experiments, indicate the kinetics of MEK and ERK phosphorylation in different cell lines or primary cells pretreated with either DMSO or 25 µM MG132 for 6 h and then stimulated with 1 nM PDGF-BB for the indicated duration in minutes. Total levels of MEK, ERK, and β-actin are not affected by MG132 treatment and serve as loading controls. Quantification of relative pMEK and pERK levels, normalized by total MEK and total ERK, respectively, are shown below (mean ± s.e.m., *n* = 3). The cell cultures tested were: *a*, NIH 3T3 fibroblasts; *b*, primary MEFs; *c*, HT-1080 human fibrosarcoma. The indicated *p* value for each time course is from two-way ANOVA analysis comparing MG132-treated and control measurements.

## Results

### Reduced Growth Factor-stimulated ERK Phosphorylation in MG132-treated Cells is Partially Attributable to Inhibition of the Upstream Cascade

Given the potentially broad-based effects of proteasome inhibition on intracellular signaling, we hypothesized that the observed reduction of ERK phosphorylation in MG132-treated cells is not caused solely by upregulation of DUSPs. Indeed, we found that many key readouts of PDGF-stimulated signaling are systemically reduced in NIH 3T3 fibroblasts pretreated with 25 µM MG132 for 6 hours (Fig. 1a). Furthest upstream is the tyrosine phosphorylation of PDGF receptors; MG132 treatment significantly reduced phosphorylated Tyr^751^ of PDGF β-receptor, a major phosphorylation site that contributes to the recruitment of phosphoinositide 3-kinase (PI3K), in a PDGF dose-dependent manner (Fig. 1b). By contrast, PDGF-stimulated phosphorylation of Akt on the activating site Ser^473^, a readout of pro-survival downstream of PI3K, is significantly reduced in MG132-treated cells at both low and high PDGF concentrations (Fig. 1c); total Akt levels were not perturbed by MG132 treatment (Fig. 1a). This suggests that, whereas the ability to recruit PI3K in cells stimulated with a subsaturating PDGF concentration is not affected by MG132 treatment, the ability to maintain Akt phosphorylation is reduced. A reduction in the catalytic activity (or expression) of PI3K or enhancement of Akt dephosphorylation can explain this result. The Akt phosphorylation kinetics for the high PDGF dose are consistent with this interpretation; stimulated phospho-Akt levels in control cells are at all times maintained at higher levels than in MG132-treated cells (Fig. 1c), despite the eventual decay of PDGF receptor phosphorylation in control cells below the levels achieved at earlier times in MG132-treated cells (Fig. 1b).

The kinetics of MEK and ERK phosphorylation on activating sites (Fig. 1d&e) follow analogous patterns to those of PDGF receptor and Akt, respectively; phosphorylation of ERK1/2, but not of MEK1/2, is significantly reduced in MG132-treated versus control cells stimulated with the low PDGF dose, whereas phosphorylation of both MEK1/2 and ERK1/2 are dramatically reduced in MG132-treated cells stimulated with the high PDGF dose. Although it would appear that MEK1/2 phosphorylation stimulated at low PDGF concentration is minimally perturbed by MG132 treatment (Fig. 1d), it should be noted that total MEK1/2 levels are modestly increased in MG132-treated cells (Fig. 1d); furthermore, the accompanying ablation of ERK1/2 activation (Fig. 1e) is expected to relieve a potent negative feedback affecting MEK1/2 phosphorylation in these cells [16]. A qualitatively similar pattern of MEK and ERK phosphorylation was found in FGF-stimulated cells (Fig. 2a&b), except that the effect of MG132 treatment on ERK phosphorylation elicited by a low dose of FGF-2 is not statistically significant by two-way ANOVA. The interpretation is that, while the MEK and ERK phosphorylation kinetics are certainly consistent with upregulation of ERK dephosphorylation activity in MG132-treated cells, suppression of ERK signaling is also affected by reduced activation of the upstream kinase(s).

### The Relationship between MEK and ERK Phosphorylation in MG132-treated Cells is Consistent with a Certain Fold-enhancement of ERK Phosphatase Activity

Based on the suggestion that MEK activation is reduced in combination with increased ERK dephosphorylation activity in MG132-treated cells, we sought to parse these two effects quantitatively. To accomplish this, we devised a kinetic modeling scheme (Fig. 3). Given the potentially complex effects of MG132 treatment on growth factor receptor-mediated signaling upstream of ERK1/2, our strategy was to fit each MEK1/2 phosphorylation time course (low/high PDGF and with/without MG132 pretreatment) to an empirical function (Fig. 3a), which serves then as the input to a modified Michaelis-Menten model of ERK phosphorylation and dephosphorylation on its two activating sites. In the case of the time courses with MG132, this model tests the consistency of the simplest hypothesis: that the phosphatase activity (intracellular concentration(s) of the enzyme(s) catalyzing dephosphorylation of the two sites on ERK1/2) is enhanced by a constant factor, while the rest of the parameters affecting ERK phosphorylation kinetics (given phosphorylated MEK kinetics as the input) were constrained to have the same values in MG132-treated and control cells.

This model was iteratively fit to the ERK data set by Monte Carlo sampling of the model parameters to obtain a large ensemble of parameter sets (*n* = 10^4^) that produce nearly equivalent qualities of fit, allowing us to evaluate the degree to which each parameter was properly constrained (Text S1). As a central estimate of the model output, the mean of the ensemble is quantitatively consistent with the corresponding ERK phosphorylation data (Fig. 3b). The corresponding estimate of the fold-upregulation of ERK phosphatase activity in MG132-treated cells is 3.61±0.15 (mean ± s.d.). The small coefficient of variation (4%) indicates that this parameter was tightly constrained by the data.

### MG132 Treatment Elicits Upregulation of Dual-specificity Phosphatases MKP1 and MKP3

Our computational analysis supports a hypothetical model whereby MG132 treatment reduces ERK phosphorylation by both reducing MEK activation and enhancing ERK dephosphorylation. Hence, we sought to confirm that DUSPs implicated in ERK1/2 dephosphorylation, such as DUSP1/MKP1 and especially DUSP6/MKP3 [9], are upregulated in our MG132-treated cells. The effects of MG132 on basal and growth factor-modulated levels of DUSP expression were found to depend on the treatment time (Fig. 4), consistent with the time scale of protein synthesis and turnover. As reported previously, a 30-minute pretreatment with MG132 was insufficient to alter the basal MKP1 levels, but after an additional 2 hours of MG132 treatment in the presence of PDGF (during which time MKP1 expression is upregulated in response to stimulation [20]), MKP1 protein levels were increased by roughly 2-fold relative to PDGF without MG132 [16]. The modulation in MG132-treated cells is consistent with reduced proteasomal degradation of MKP1. For MKP3, the 30-minute MG132 pretreatment had no apparent effect on MKP3 expression before or after PDGF treatment, whereas 2- and 6-hour pretreatments with MG132 resulted in progressive upregulation of both MKP1 and MKP3 (Fig. 4a&b). With 6-hour MG132 pretreatment, both basal and PDGF-stimulated expression levels are consistently elevated, although the overall elevation of MKP3 is not statistically significant at the *p* = 0.05 level (two-way ANOVA); this is attributed to the shape of the MKP3 time course, which dips down at early stimulation times, bringing MKP3 expression in MG132-treated cells down to a level that is similar to those in control cells at time zero and at time = 120 minutes (Fig. 4b).

Although MKP1 and MKP3 are upregulated in MG132-treated cells, to an extent that can explain the apparent decrease in MEK-catalyzed ERK phosphorylation, we previously found no correlation between the expression levels of these particular DUSPs and the kinetics of growth factor-stimulated ERK phosphorylation [16]; however, these results point to the possibility that other DUSPs, or/and other phosphatases capable of dephosphorylating either of the two activating sites on ERK, are upregulated to a similar extent in MG132-treated cells.

### The Effects of MG132 Treatment on MEK and ERK Phosphorylation Vary Across Cell Backgrounds

To partially test the generality of the results reported here, we evaluated the effects of MG132 treatment on PDGF-stimulated MEK and ERK phosphorylation in NIH 3T3 fibroblasts as before, alongside parallel measurements for primary mouse embryonic fibroblasts (MEFs) and HT-1080 human fibrosarcoma cells (Fig. 5). In the NIH 3T3 line, both MEK and ERK phosphorylation levels again showed partial inhibition as a consequence of MG132 treatment (Fig. 5a), whereas in MEFs there was partial reduction of ERK phosphorylation but no discernible reduction in MEK phosphorylation kinetics in MG132-treated cells (Fig. 5b). In the transformed HT-1080 cell line, PDGF stimulates MEK and ERK phosphorylation above the already elevated basal level mediated by oncogenic N-Ras [21]. As in NIH 3T3 cells, both MEK and ERK phosphorylation in HT-1080 cells showed apparent sensitivity to MG132 treatment, although we note that the measured MEK phosphorylation response showed little change from the basal level, and therefore the overall effect of MG132 is not statistically significant (Fig. 5c). We conclude that although growth factor-stimulated ERK phosphorylation was muted by MG132 treatment in all three mesenchymal cell backgrounds tested, the mode of regulation manifest at the level of MEK phosphorylation exhibits differential sensitivity to proteasome inhibition.

## Discussion

Pharmacological inhibitors vary in both promiscuity and breadth of biological outcomes. An inhibitor might antagonize multiple molecular targets, or it might act on a quite narrow range of targets that nonetheless mediate pleiotropic effects. Broad effects should be expected in cells treated with a proteasome inhibitor, irrespective of its specificity. The ubiquitin-proteasome degradation pathway clearly shows some degree of selectivity in regulating the expression levels of protein targets, but it nonetheless impacts a broad range of signal transduction pathways and other intracellular processes.

In NIH 3T3 fibroblasts, it was found that proteasome inhibition by treatment with MG132 reduced receptor tyrosine kinase-mediated signal transduction at multiple nodes of the network. In PDGF-stimulated cells, phosphorylation of the PDGF β-receptor on Tyr^751^ was reduced, as were the phosphorylation levels of the downstream kinases Akt, MEK, and ERK. A plausible explanation for these findings is that multiple phosphatases are upregulated in proteasome-inhibited cells. In endothelial cells, proteasome inhibition has been shown to upregulate the serine-threonine phophatase PP2A, accompanied by reduced Akt phosphorylation [22]. It is well known that PP2A also dephosphorylates Raf and MEK isoforms; however, we checked for upregulation of each of the three PP2A subunits in MG132-treated NIH 3T3 cells and found no discernible change in abundance (results not shown). Whereas activating sites on Akt and MEK are dephosphorylated by serine-threonine phosphatases, upregulation of one or more protein-tyrosine phosphatases might explain the reduction in PDGF receptor phosphorylation, which apparently more than compensates for any tempering of Cbl-mediated receptor turnover [23], [24] resulting from MG132 treatment. If so, the lack of significant effect on PDGF receptor Tyr^751^ phosphorylation at the low PDGF dose (Fig. 1b) might be attributed to protection of the site by the saturable, high avidity interaction of the PI3K regulatory subunit [25]. Other possible negative regulators of the Ras-ERK pathway that are subject to proteasomal degradation include Sprouty/Spred-family proteins [26], [27].

The complexity of ERK modulation by proteasome inhibition, considering the direct and indirect effects on ERK phosphorylation status and the dynamic nature of the pathway, demands a quantitative analysis. We contend that kinetic modeling is a useful approach for parsing multiple, time-dependent effects on biochemical systems. A key step in its implementation is choosing the degree of model complexity, since the mathematical description of a system’s mechanistic details comes with the need to specify a certain number of rate parameters, which might or might not be appropriate depending on the availability of quantitative data [28]. The data here allowed a reasonably mechanistic description of ERK phosphorylation and dephosphorylation kinetics, based on the common assumption that the kinase activity (*V_max_*) of MEK on ERK is directly proportional to the measured level of phosphorylated MEK; in turn, this allowed the evaluation of the postulated upregulation of ERK phosphatase activity. In contrast, it was not prudent to attempt to model in mechanistic detail the multiple effects of proteasome inhibition affecting the kinetics of MEK phosphorylation. Thus, consideration of the mechanistic uncertainties in constructing such a mathematical model can serve as a guide as to which research questions might be addressed given the data in hand.

## Supporting Information

Text S1 Details of Computational Methods.
(PDF)

## References

[1] Muratani M, Tansey WR (2003) How the ubiquitin-proteasome system controls transcription. Nat Rev Mol Cell Biol 4: 192–201. 12612638 10.1038/nrm1049

[2] Glickman MH, Ciechanover A (2002) The ubiquitin-proteasome proteolytic pathway: Destruction for the sake of construction. Physiol Rev 82: 373–428. 11917093 10.1152/physrev.00027.2001

[3] Adams J (2004) The proteasome: A suitable antineoplastic target. Nat Rev Cancer 5: 349–360. 10.1038/nrc1361 15122206

[4] Yu CR, Rahmani M, Dent P, Grant S (2004) The hierarchical relationship between MAPK signaling and ROS generation in human leukemia cells undergoing apoptosis in response to the proteasome inhibitor Bortezomib. Exp Cell Res 295: 555–566. 15093752 10.1016/j.yexcr.2004.02.001

[5] Shaul YD, Seger R (2007) The MEK/ERK cascade: From signaling specificity to divers functions. Biochim Biophys Acta Mol Cell Res 1773: 1213–1226. 10.1016/j.bbamcr.2006.10.005 17112607

[6] Yoon S, Seger R (2006) The extracellular signal-regulated kinase: Multiple substrates regulate diverse cellular functions. Growth Factors 24: 21–44. 16393692 10.1080/02699050500284218

[7] Dhillon AS, Hagan S, Rath O, Kolch W (2007) MAP kinase signalling pathways in cancer. Oncogene 26: 3279–3290. 17496922 10.1038/sj.onc.1210421

[8] Roberts PJ, Der CJ (2007) Targeting the Raf-MEK-ERK mitogen-activated protein kinase cascade for the treatment of cancer. Oncogene 26: 3291–3310. 17496923 10.1038/sj.onc.1210422

[9] Owens DM, Keyse SM (2007) Differential regulation of MAP kinase signalling by dual-specificity protein phosphatases. Oncogene 26: 3203–3213. 17496916 10.1038/sj.onc.1210412

[10] Lin YW, Chuang SM, Yang JL (2003) ERK1/2 achieves sustained activation by stimulating MAPK phosphatase-1 degradation via the ubiquitin-proteasome pathway. J Biol Chem 278: 21534–21541. 12676937 10.1074/jbc.M301854200

[11] Lornejad-Schafer MR, Schafer C, Richter L, Grune T, Haussinger DH, et al (2005) Osmotic regulation of MG-132-induced MAP-kinase phosphatase MKP-1 expression in H4IIE rat hepatoma cells. Cell Physiol Biochem 16: 193–206. 16301819 10.1159/000089845

[12] Chan DW, Liu VWS, Tsao GSW, Yao K-M, Furakawa T, et al (2008) Loss of MKP3 mediated by oxidative stress enhances tumorigenicity and chemoresistance of ovarian cancer cells. Carcinogenesis 29: 1742–1750. 18632752 10.1093/carcin/bgn167

[13] Jurek A, Amagasaki K, Gembarska A, Heldin C-H, Lennartsson J (2009) Negative and positive regulation of MAPK phosphatase 3 controls platelet-derived growth factor-induced Erk activation. J Biol Chem 284: 4626–4634. 19106095 10.1074/jbc.M808490200

[14] Lønne GK, Masoumi KC, Lennartsson J, Larsson C (2009) Protein kinase Cδ supports survival of MDA-MB-231 breast cancer cells by suppressing the ERK1/2 pathway. J Biol Chem 284: 33456–33465. 19833733 10.1074/jbc.M109.036186 PMC2785190

[15] Kucharska A, Rushworth LK, Staples C, Morrice NA, Keyse SM (2009) Regulation of the inducible nuclear dual-specificity phosphatase DUSP5 by ERK MAPK. Cell Signalling 21: 1794–1805. 19666109 10.1016/j.cellsig.2009.07.015

[16] Cirit M, Wang C-C, Haugh JM (2010) Systematic quantification of negative feedback mechanisms in the extracellular signal-regulated kinase (ERK) signaling network. J Biol Chem 285: 36736–36744. 20847054 10.1074/jbc.M110.148759 PMC2978602

[17] Pera MF, Filipczyk AA, Hawes SM, Laslett AL (2003) Isolation, characterization, and differentiation of human embryonic stem cells. Methods Enzymol 365: 429–446. 14696363 10.1016/s0076-6879(03)65030-5

[18] Wang C-C, Cirit M, Haugh JM (2009) PI3K-dependent crosstalk interactions converge with Ras as quantifiable inputs integrated by Erk. Mol Syst Biol 5: article no.246. 10.1038/msb.2009.4 PMC2657535 19225459

[19] Cirit M, Haugh JM (2012) Data-driven modelling of receptor tyrosine kinase signalling networks quantifies receptor-specific potencies of PI3K- and Ras-dependent ERK activation. Biochem J 441: 77–85. 21943356 10.1042/BJ20110833 PMC3687362

[20] Brondello J, Brunet A, Pouysségur J, McKenzie FR (1997) The dual specificity mitogen-activated protein kinase phosphatase-1 and -2 are induced by the p42/p44^MAPK^ cascade. J Biol Chem 272: 1368–1376. 8995446 10.1074/jbc.272.2.1368

[21] Hall A, Marshall CJ, Spurr NK, Weiss RA (1983) Identification of transforming gene in two human sarcoma cell lines as a new member of the ras gene family located on chromosome 1. Nature 303: 396–400. 6304521 10.1038/303396a0

[22] Wei Q, Xia Y (2006) Proteasome inhibition down-regulates endothelial nitric-oxide synthase phosphorylation and function. J Biol Chem 281: 21652–21659. 16737962 10.1074/jbc.M602105200

[23] Miyake S, Mullane-Robinson KP, Lill NL, Douillard P, Band H (1999) Cbl-mediated negative regulation of platelet-derived growth factor receptor-dependent cell proliferation: A critical role for Cbl tyrosine kinase-binding domain. J Biol Chem 274: 16619–16628. 10347229 10.1074/jbc.274.23.16619

[24] Chiarugi P, Cirri P, Taddei ML, Talini D, Doria L, et al (2002) New perspectives in PDGF receptor downregulation: the main role of phosphotyrosine phosphatases. J Cell Sci 115: 2219–2232. 11973362 10.1242/jcs.115.10.2219

[25] Kazlauskas A, Cooper JA (1990) Phosphorylation of the PDGF receptor β-subunit creates a tight binding site for phosphatidylinositol-3 kinase. EMBO J 9: 3279–3286. 2170111 10.1002/j.1460-2075.1990.tb07527.x PMC552064

[26] Hall AB, Jura N, DaSilva J, Jang YJ, Gong D, et al (2003) hSpry2 is targeted to the ubiquitin-dependent proteasome pathway by c-Cbl. Curr Biol 13: 308–314. 12593796 10.1016/s0960-9822(03)00086-1

[27] Lock P, I ST, Straffon AFL, Schieb H, Hovens CM, et al (2006) Spred-2 steady-state levels are regulated by phosphorylation and Cbl-mediated ubiquitination. Biochem Biophys Res Comm 351: 1018–1023. 17094949 10.1016/j.bbrc.2006.10.150

[28] Cirit M, Haugh JM (2011) Quantitative models of signal transduction networks: How detailed should they be? Commun Integr Biol 4: 353–356. 21980579 10.4161/cib.4.3.15149 PMC3187907

