# Supplementary material for: Systemic Perturbation of the ERK Signaling Pathway by the Proteasome Inhibitor, MG132
Source: PLoS One. 2012 Nov 30;7(11):e50975. doi: 10.1371/journal.pone.0050975 (PMC3511445; doi:10.1371/journal.pone.0050975)
Supplement: Text S1 — Details of Computational Methods. (PDF) [file pone.0050975.s001.pdf]

Systemic perturbation of the ERK signaling pathway by the proteasome inhibitor, MG132

Murat Cirit, Kyle G. Grant, and Jason M. Haugh

Supplementary Text S1: Details of Computational Methods

Empirical fit of MEK phosphorylation kinetics

Given the potentially complex effects of MG132 treatment on growth factor receptor-mediated signaling upstream of ERK1/2, our strategy was to fit each of the four MEK1/2 phosphorylation time courses (low/high PDGF and with/without MG132 pretreatment) to an empirical function. Inspection of the data suggested that, as a minimal description, a function with two time exponentials and four adjustable parameters would be sufficient. The variable *ppMEK*(*t*) represents dually phosphorylated (active) MEK as a function of time, *t*.

$$ppMEK(t) = A_0 + A_1 e^{-k_1 t} - \left(A_0 + A_1\right) e^{-k_2 t}. \qquad \text{(Eq. S1)}$$

The phenomenological parameters A_0, A_1, k_1, and k_2 are determined by nonlinear least-squares fitting, with different values for each time course. The fits are constrained so that all of these parameters are positive. Roughly speaking, A_0 is the plateau level reached at long times, A_1 primarily determines the level of the peak and thus the magnitude of the adaptation, and the rate constants k_1 and k_2 collectively determine the shape of the temporal response (how quickly the peak is achieved and the rate of relaxation to A_0). As in our previous models, we assume that the basal MEK phosphorylation level is negligible; i.e., Eq. S1 has the property *ppMEK*(0) = 0.

The fits to the data are presented in Fig. 3a of the paper, with fit parameters given in the table below. The data were fit directly, without further normalization, and therefore the parameters A_0 and A_1 are dimensionless and set on the same arbitrary scale as the data (which are ratios of two densitometric measurements).

Parameter	***DMSO, 0.03 nM PDGF***	***DMSO, 1 nM PDGF***	***MG132, 0.03 nM PDGF***	***MG132, 1 nM PDGF***
A_0	0.00687	0.0188	0.000798	0.000709
A_1	0.0325	0.0732	0.664	0.878
k_1 (min^{-1})	0.0576	0.0376	0.107	0.112
k_2 (min^{-1})	0.242	1.04	0.115	0.122

Kinetic model of ERK phosphorylation

With each empirical *ppMEK*(*t*) function as an input, the next step is to develop a mechanistic model of ERK phosphorylation using globally fit kinetic parameters. The fractions of total ERK in the non-phosphorylated, mono-phosphorylated, and dual-phosphorylated forms are defined as *ERK*, *pERK*, and *ppERK*, respectively, and conservation equations are constructed in modified Michaelis-Menten form; it is assumed that the ERK species equilibrate rapidly with the enzymes that modify them and potentially compete for available enzyme.

$$\frac{dERK}{dt} = -\frac{C_1(ppMEK)(ERK)}{1 + ERK/K_1 + pERK/K_2} + \frac{C_{-1}\alpha_{MG132}pERK}{1 + pERK/K_{-1} + ppERK/K_{-2}}; \qquad \text{(Eq. S2)}$$

$$ERK(0) = 1.$$

$$\frac{dppERK}{dt} = \frac{C_2(ppMEK)(pERK)}{1 + ERK/K_1 + pERK/K_2} - \frac{C_{-2}\alpha_{MG132}ppERK}{1 + pERK/K_{-1} + ppERK/K_{-2}}; \qquad \text{(Eq. S3)}$$

$$ppERK(0) = 0.$$

$$pERK = 1 - ERK - ppERK. \qquad \text{(Eq. S4)}$$

The global fit parameters here include catalytic efficiencies C_i (scaled versions of the corresponding V_{max}/K_M ratios), and Michaelis saturation constants K_i, where subscripts i = 1/–1 and 2/–2 signify phosphorylation/dephosphorylation of the first and second phosphorylation site on ERK, respectively. The parameter α_{MG132} represents the fold-change in ERK phosphatase expression in MG132-treated cells; i.e., it is absent (or set to 1) in the fitting of the DMSO-treated control data.

With certain changes to the notation and a rescaling of C_1 and C_2 to reflect the arbitrary units of the *ppMEK*(*t*) function as used here, Eqs. S2-S4 are identical to those used previously to describe the phosphorylation of ERK [1]. They are also related to equations used in earlier data-driven models [2,3], the only change being that the amount of ERK phosphatase enzyme is taken here to be constant in time. The justification for this simplification was two-fold: the best fit of the previous PDGF receptor network model was achieved with constant ERK phosphatase activity, and experimentally we found no relationship between the expression levels of MKP1 and MKP3 (dual-specificity phosphatases that respond in different ways to growth factor stimulation) and ERK phosphorylation [3].

Summary of model parameters and global fitting to ERK phosphorylation data

The 8 constant parameters invoked in equations Eqs. S2-S4 above were subjected to a global fit to the phospho-ERK data set. A large ensemble of parameter sets was obtained using a modified simulated annealing algorithm described in detail previously [3]. These were sorted according to the lowest sum of squared deviations (*SSD*, or χ^2) to identify the 10,000 "best" parameter sets. As explained previously [2,3], each parameter set is associated with a conversion factor that best aligns the dimensionless variables of the model to the arbitrary instrument units of the phospho-ERK data. The central estimate of the model is expressed as the mean of the aligned outputs (n = 10,000), which is recomputed once the parameter set ensemble is identified.

Information about the distributions of parameter values in the ensemble is provided in the table below. This analysis shows that, among the parameters, the degree of ERK phosphatase upregulation in MG132-treated cells (α_{MG132}) is especially well constrained. By comparison, most of the other parameters vary by an order of magnitude or more. This can be attributed to the fact that the absolute stoichiometry of ERK phosphorylation is not specified, whereas the phospho-ERK levels in MG132-treated cells relative to control cells is directly addressed in the data.

Parameter	*Minimum*	*Lower quartile*	*Median*	*Upper quartile*	*Maximum*
C_1 (min^{-1})	276	789	1.12e3	1.58e3	3.35e4
K_1	0.795	4.51	5.39	7.73	10.2
C_{-1} (min^{-1})	0.210	0.317	0.365	0.426	0.716
K_{-1}	2.22	9.28	11.8	14.8	21.5
C_2 (min^{-1})	7.2e3	1.9e4	2.4e4	3.3e4	6.8e4
K_2	7.5e-4	1.5e-3	2.2e-3	3.8e-3	7.2e-3
C_{-2} (min^{-1})	3.00	6.13	11.6	16.6	24.2
K_{-2}	4.11	38.1	60.7	82.4	157
α_{MG132}	2.84	3.51	3.61	3.71	4.11

References

[1] Cirit, M. and Haugh, J.M. (2012). Data-driven modelling of receptor tyrosine kinase signalling networks quantifies receptor-specific potencies of PI3K- and Ras-dependent ERK activation. Biochem. J. 441, 77-85.

[2] Wang, C.-C., Cirit, M. and Haugh, J.M. (2009). PI3K-dependent crosstalk interactions converge with Ras as quantifiable inputs integrated by Erk. Mol. Syst. Biol. 5, article no. 246.

[3] Cirit, M., Wang, C.-C. and Haugh, J.M. (2010). Systematic quantification of negative feedback mechanisms in the extracellular signal-regulated kinase (ERK) signaling network. J. Biol. Chem. 285, 36736-36744.
